# Reelin immunoreactivity in neuritic varicosities in the human hippocampal formation of non-demented subjects and Alzheimer’s disease patients

**DOI:** 10.1186/2051-5960-1-27

**Published:** 2013-06-26

**Authors:** Tina Notter, Irene Knuesel

**Affiliations:** 1 Institute of Pharmacology and Toxicology, University of Zurich, Winterthurerstrasse 190, Zurich, CH-8057, Switzerland

**Keywords:** Reelin, Axonal and dendritic varicosities, Hippocampal formation, Aging, Alzheimer’s disease

## Abstract

**Background:**

Reelin and its downstream signaling members are important modulators of actin and microtubule cytoskeleton dynamics, a fundamental prerequisite for proper neurodevelopment and adult neuronal functions. Reductions in Reelin levels have been suggested to contribute to Alzheimer’s disease (AD) pathophysiology. We have previously reported an age-related reduction in Reelin levels and its accumulation in neuritic varicosities along the olfactory-limbic tracts, which correlated with cognitive impairments in aged mice. Here, we aimed to investigate whether a similar Reelin-associated neuropathology is observed in the aged human hippocampus and whether it correlated with dementia status.

**Results:**

Our immunohistochemical stainings revealed the presence of N- and C-terminus-containing Reelin fragments in corpora amylacea (CAm), aging-associated spherical deposits. The density of these deposits was increased in the molecular layer of the subiculum of AD compared to non-demented individuals. Despite the limitation of a small sample size, our evaluation of several neuronal and glial markers indicates that the presence of Reelin in CAm might be related to aging-associated impairments in neuronal transport leading to accumulation of organelles and protein metabolites in neuritic varicosities, as previously suggested by the findings and discussions in rodents and primates.

**Conclusions:**

Our results indicate that aging- and disease-associated changes in Reelin levels and proteolytic processing might play a role in the formation of CAm by altering cytoskeletal dynamics. However, its presence may also be an indicator of a degenerative state of neuritic compartments.

## Background

The increase in human life expectancy has entailed a pronounced rise in age-associated neurodegenerative diseases with the most prominent form being Alzheimer’s Disease (AD), affecting over 26 million people worldwide [1]. Despite extensive research performed during the past, no efficient therapies for this progressive brain disease are currently available. Most of the scientific approaches were based on the “amyloid cascade hypothesis” that placed the β- and γ-secretase-generated amyloid-β peptides as causal factors of the pathophysiology [2,3]. While this may hold true for the familial form of AD with its dominant mutations in either the amyloid precursor protein (*APP*, [4,5]) or presenilin 1 and 2 (*PSEN*; [6,7]) genes, the initiating trigger of the aging-associated, sporadic form of AD is still unknown. Based on recent experimental evidence demonstrating that multiple exposures to viral-like immune challenges are sufficient to induce AD-like neuropathology in aged wild-type mice [8], we were able to identify a crucial trigger and to reconstruct the temporal-spatial sequence of pathophysiological changes that characterize the earliest disease stages. The integration of experimental data of the last three decades enabled us to substantiate and expand our findings to propose a cellular mechanism of the neuropathological changes and its progression across interconnected brain areas that characterize sporadic AD [9,10]. The new hypothesis highlights the detrimental effect of chronic inflammatory conditions on basic cellular functions in long-projection neurons of the olfactory-limbic system during aging. The selective vulnerability of these neurons includes inflammation-induced impairments in the Reelin-mediated signaling pathway across these fundamental neuronal networks [10]. By binding to the apolipoprotein E receptor 2 (ApoER2) and the very low density lipoprotein receptor (VLDLR) [11,12], the extracellular matrix protein Reelin regulates cytoskeletal dynamics essential for migrating neurons [13,14], developing neurites [15,16] and adult spines [17,18]. The signaling pathway involves cytosolic cascades that ultimately inhibit the major tau kinases, Glycogen Synthase Kinase 3β (GSK3β) and Cyclin-Dependent Kinase 5 (CDK5) [19-21], as well as activates the LIM1 kinase and increases n-cofilin phosphorylation [22] to modulate both the microtubule and actin cytoskeleton, respectively. Engaging the same signaling pathway, Reelin also exerts a crucial role at adult synaptic sites by modulating NMDA receptor functions [23-27]. Recent experimental evidence further highlighted that Reelin maintains synaptic plasticity by competing with ApoE4 to prevent the latter from sequestering NMDA, AMPA, and ApoER2 receptors in intracellular compartments [27]. In line with the well described aging-associated synaptic impairments, Reelin expression has been shown to decline in aging rodents, correlating with hippocampus-dependent learning and memory performance [28,29]. Furthermore, Reelin accumulates within neuritic varicosities in both rodents and non-human primates [28,30], possibly related to the aging associated decrease in Reelin signaling and its protective modulation of cytoskeleton dynamics [10]. In agreement with these experimental data, recent genome wide association studies performed in elderly, non-demented individuals identified three single nucleotide polymorphisms (SNPs) in the Reelin gene that significantly correlated with increased Tau phosphorylation and concomitant appearance of neurofibrillary tangles (NFTs) [31]. Moreover, immunohistochemical investigations involving human brains revealed significantly decreased levels of Reelin in patients with mild cognitive impairments (MCI) and AD compared to non-demented subjects [32,33]. Further evidence of altered Reelin-mediated signaling in AD was provided by genetic [34] and biochemical studies [35,36] suggesting that decreased Reelin production may contribute to the initiation and progression of AD by impairing synaptic functions, cytoskeleton stability and proper axonal transport. Interestingly, alterations in Reelin glycosylation, its proteolytic cleavage and degradation, as well as changes in its mRNA stability were shown to be changed in brain homogenates of AD patients [35,36], further highlighting the importance of proper Reelin signaling during aging.

Here, we investigated the localization and levels of Reelin in the postmortem human brain by selectively assessing its putative accumulation in neuritic varicosities in non-demented elderly and patients with AD by immunohistochemistry and unbiased stereological analyses. Complementary immunoblotting of corresponding CSF samples was performed to examine potential alterations in Reelin proteolytic processing.

## Methods

### Human tissue

Formalin fixed paraffin-embedded hippocampal brain tissue blocks of eight AD patients and eight age-matched non-demented individuals were obtained from the Netherland Brain Bank, Amsterdam, Netherlands (Table 1). Blocks were de- and re-paraffinized with fresh paraffin. Serial sections (5 μm) were cut on a sliding microtome with an inter-section interval of 100 μm. Cerebral spinal fluid (CSF, obtained postmortem from the lateral ventricles with an 18GA 3.50 inch spinal needle) of each subject was thawed and centrifuged at 20’000 rpm for 15 minutes at 4°C, the supernatant aliquoted and stored at −80°C until further use. Quality assessments revealed no blood contaminations in the CSF samples. Additional test tissue for qualitative investigations included paraffin-embedded sections (cut at 10 μm) from the hippocampus of 6 non-demented and 6 age-matched AD patients (kindly provided by Professor Manuela Neumann, German Center for Neurodegenerative Diseases (DZNE) Tübingen and Institute of Pathology and Neuropathology).

**Table 1:** Details of human postmortem brain tissue included in the analyses

**NBB-Code**	**Sex**	**Age (years)**	**PMD (h.min)**	**Brain weight (g)**	Braak stages*	**Apoe genotype**	**Clinicopathological data**	**pH (csf)**	**[Protein]mg/ml**
**Controls**									
07-082^§^	M	81	7.55	1194	2 0	3/3	Renal insufficiency and decompensatio cordis	6.23	4.6
00-142	F	82	5.30	1260	1 A	3/2	Myocardial infarct	6.60	3.14
03-061	F	83	5.30	1274	1 B	3/2	Squamous cell carcinoma of the left maxilla	6.48	3.62
05-083	F	85	5.00	1217	1 B	3/3	Multi organ failure after a ruptured abdominal aneurysm	6.72	4.46
98-157^§^	M	85	5.13	1383	2 A	3/2	Cardiac tamponade	6.23	2.92
04-061	F	88	6.15	1121	? B	3/3	Old age	6.98	2.79
96-105^§^	M	88	5.40	1120	4	4/3	Cardiac arrest	6.65	1.4
96-044^§^	F	90	5.50	1046	2 A	3/3	unknown	7.00	2.79
**AD**									
06-013^§^	M	81	4.50	1193	4 C	4/4	Dehydration, sepsis?	6.42	3.02
09-105	F	82	5.25	999	5 C	n/a	Heart failure	6.08	3.63
00-138^§^	F	84	5.15	1098	5 C	3/3	Pneumonia with dehydration	6.65	2.2
97-093	M	86	5.35	1250	5 C	4/3	CVA/Myocardial infarction	6.39	1.93
06-044	F	86	5.55	930	4 B	4/3	Cachexia	6.85	1.8
04-064	F	88	6.25	1079	5 C	3/3	Organ failure due to dehydration	6.60	2.88
97-003^§^	M	88	5.10	984	5 B	4/3	Pneumonia	6.45	2.38
96-049	F	90	3.50	925	5	4/3	Dehydration	7.02	2.81

*According to [37,38].

PMD = postmortem delay.

^§^ Tissue blocks containing hippocampus and EC.

### Antibodies

The following antibodies were used: mouse monoclonal anti-Reelin recognizing the N-terminus (clone G10, MAB 5364, Milipore 1:200 and clone 142, MAB 5366, Milipore 1:200); mouse monoclonal anti-Reelin antibodies (C-terminal subrepeats 8A and 8B, 1:200 each, kindly provided by Professor André Goffinet, University of Louvain Medical School, Brussels, Belgium); rabbit polyclonal anti-amyloid β (1-40/42), (AB5076; Millipore, 1:200); mouse monoclonal anti APP-A4, clone 22C11 (MAB348 Millipore, 1:500), mouse monoclonal anti-PHF Tau (clone AT100, MN1060; Thermo Scientific, 1:1000); polyclonal rabbit anti-Tau pS422 (44-764G, Invitrogen,1:1000); rabbit polyclonal anti-GFAP (Z0334, DAKO, 1:2500); mouse monoclonal anti-GFAP (MAB 360, Millipore, 1:10’000); rabbit polyclonal anti-Iba1 (019–19741, Wako Pure Chemical, 1:2000); polyclonal rabbit anti-MAP2(26*) (microtubule-associated protein 2) (AB5622, Chemicon, 1:2000); polyclonal rabbit anti-Synapsin-1 (A-6442, Molecular Probes, 1:1000); polyclonal rabbit anti-Synaptophysin (A010, DAKO, 1:500); rabbit polyclonal anti-α-Synuclein (ab52168, Abcam, 1:250); mouse monoclonal anti-CD 45 (M0701, 1:100, kindly provided by Prof. Karl Frei, Department of Neurosurgery, University Hospital Zürich, Switzerland).

### Immunohistochemistry

#### Immunoperoxidase staining

Sections were deparaffinized in xylol (3 times for 3 minutes), rehydrated in decreasing ethanol concentrations (twice 100%, 96%, 70% and twice dH_2_O for 3 minutes each) and washed in 50 mM Tris-saline, pH 7.4 (1×Tris). The following antigen retrieval techniques were applied: two 5-minute citrate buffer microwave irradiations at 800 W (to visualize Reelin-producing cells with anti-Reelin 142 antibody, see Additional file 1: Figure S1), 10-minute citrate buffer microwave irradiation at 80°C, either followed by a 10-minute pepsin treatment [39] or not, and a 5-minute 95% formic acid treatment (to visualize Aβ plaques). Furthermore, a 10-minute 3% H_2_O_2_/methanol treatment was used to block endogenous peroxidase reactivity. Sections were then incubated for 30 min in blocking solution (1×Tris containing 5% normal horse serum, 5% normal goat serum, 4% of BSA), transferred to the primary antibody solution (diluted in 1×Tris, containing 2.5% normal horse serum, 2.5% normal goat serum, 2% BSA) for overnight incubation at 4°C. Sections were washed in 1×Tris and incubated for 30 minutes with biotinylated secondary antibodies (1:200 in same solution as used for primary antibodies). After 3 washes in 1×Tris, sections were processed for the DAB immunoperoxidase using the Avidin-Peroxidase-Complex (ABC, Vectastain Elite kit) staining techniques as described previously [40-42]. To visualize cell nuclei and corpora amylacea (CAm), sections were counterstained with Harry's Hematoxylin (HHS-128, Sigma-Aldrich; very weak staining of CAm) or Ehrlich’s Hematoxylin (100 ml dH_2_O, 100 ml 96% ethanol, 100 ml glycerol, 10 ml glacial acetic acid, 2 g hematoxylin, 3 g kalialaun, 0.4 g sodium jodate; strongly reacts with CAm [43], kindly provided by Charlotte Burger, University of Zurich, Institute for Anatomy). In brief, sections were incubated for 2 minutes in the Hematoxylin solution, rinsed in tap water, differentiated in acetic alcohol and blued in running tap water for 10 minutes, dehydrated and coverslipped with Eukitt (Erne Chemie). Toloidinblue staining was performed according to standard protocols.

#### Immunofluorescence staining

The protocol was identical to the described immunoperoxidase staining with the following changes: All washing steps and antibody dilutions were done in phosphate-buffered saline (PBS) at pH 7.4. Sections were blocked for 1 hour and incubated for 45 min with the secondary antibodies (coupled to either Alexa Fluor488 or Cy3 (dilution 1:1000 and 1:500, respectively; Molecular Probes, Invitrogen). To reduce lipofuscin-associated auto-fluorescence in aged cells, sections were incubated in 0.1% Sudan Black (Carl Roth GmbH) dissolved in 70% methanol for 2 minutes, briefly washed with PBS and air-dried [44]. Sections were then coverslipped with Dako-DAPI solution (Dako) or ProLong Gold Antifade reagent (Invitrogen) to visualize cell nuclei.

#### Microscopy and image acquisition

Immunoperoxidase-stained sections were scanned and visualized with an automated upright slide-scanning microscope (Zeiss) in brightfield mode and the Pannoramic Viewer 1.8.3.0 (3D Histotech Ltd), respectively. High magnification images were acquired with a brightfield light microscope (Axioscop 2, Zeiss) connected to a digital camera (AxioCam, Zeiss) and captured with AxioVision software (Version 4.5., Zeiss), or with a confocal microscope (LSM 710, Zeiss) and processed with Imaris 7.1.1 software (Bitplane Inc.) and Adobe Photoshop CS5 (Adobe) software.

### Quantitative and statistical analyses

**Figure 1:**
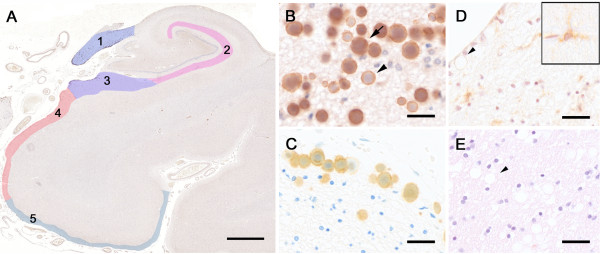
**Reelin immunoreactivity in the aged human hippocampal formation. A)** Reelin (G10 antibody) immunoperoxidase labeling in combination with hematoxylin (Ehrlich) counterstaining. The tissue section was obtained from an 88 year-old ND individual. The color-coding represents the areas included in the stereological analysis: 1=fornix, 2=stratum lacunosum moleculare (SLM), 3=molecular layer of subiculum, 4=molecular layer of pre/parasubiculum, 5=molecular layer of entorhinal cortex (EC). **B)** Higher magnification view of Reelin-positive deposits in the fornix. Arrow points to a filled deposit, arrowhead indicates a hollow deposit. **C)** Reelin (G10) immunoperoxidase labeling combined with toloidinblue counterstaining in the fornix border area. Tissue section was obtained from an 80 year-old AD patient. **D)** Control staining using isogenic IgGs (mouse anti-human CD45 antibody) following antigen retrieval (microwave irradiation in citrate buffer plus pepsin pretreatment) and Hematoxylin (Harrys) counterstaining. Brain section was obtained from a 79 year-old ND individual. Arrowhead points to a CD45-negative deposit, insert represents a CD45-positive lymphocyte associated with a brain capillary. **E)** Control staining without primary antibody following antigen retrieval and Hematoxylin/Eosin counterstaining. Brain section was obtained from an 81 year-old ND individual. Arrowhead points to an immune-negative deposit. Scale bars: **A** = 2 mm, **B** - **D** = 30 μm.

Stereological estimations of Reelin-positive CAm area and density were performed using the Stereo Investigator 10.50 software (Microbrightfield Bioscience). Two sections with an intersection distance of 200 μm were used for quantification. The following regions were included for the analyses based on the density of the deposits (Figure 1): fornix, stratum lacunosum moleculare (SLM), layer I of subiculum, layer I of pre/parasubiculum and layer I of entorhinal cortex (EC) [45,46]. Other areas of the hippocampal formation were not included to minimize measurement errors. The Cavalieri estimator with a grid spacing of 100 μm was used to estimate the area of the region of interest. For fractionator sampling, an unbiased counting frame sized between 80 and 200 μm (depending on the deposit density) was applied to estimate the Reelin-positive CAm profile density. Concomitantly, the individual CAm areas were estimated using the nucleator with 4 rays. The area fraction (AF) was calculated using the following equation: AF = N_CA_ × A_CA_/A_tot_, where N_CA_ is the estimated profile number of Reelin-positive CAm within the measured area, A_CA_ is the mean area of CAm within the measured area, and A_tot_ is the total area measured. Statistical analysis of the area and density of Reelin-positive CAm was performed using StatView software. For each brain region, a factorial ANOVA was performed with *Group* (2 levels: ND and AD) and *Deposit type* (2 levels: filled vs hollow) as independent variables, and *AF* (area fraction), *A*_*CA*_ (mean CAm size), *N*_*CA*_ (estimated number of Reelin-positive CAm) as dependent variables. Pearson’s product moment correlations were performed between Reelin-positive CAm (*AF,* filled and hollow combined) and Western blot data (full-length Reelin, NR2, NR6 and 60 kDa fragments). Statistical significance was set at p<0.05.

### Western blot analysis

Protein concentrations of the CSF samples were measured using NanoDrop (Table 1). Equal volumes of CSF were analyzed. The sample preparation was performed as previously described [47]. Western blotting was done as described [47] with the following adaptations: 3–8% Tris-Acetate Midi Gels were used (NuPAGE Novex Tris-Acetate Midi Gel). The electrophoresis was performed using the Xcell4 *SureLock* Midi-Cell system (Cat.No. WR0100, Invitrogen). Proteins were blotted onto PVDF membranes using the Novex Semi-Dry Blotter (Cat.No. SD1000, Invitrogen). Films were scanned and analyzed using ImageJ Launcher software; intensities of bands were measured (area under the curve, AUC) and quantified. Reelin immunoreactivity was statistically compared between ND and AD subjects using the non-parametrical Mann–Whitney U test using StatView software. Statistical significance was set at p<0.05. Scanned films were processed with Adobe Photoshop CS5 for visual display (Adobe software).

## Results

### Reelin immunoreactivity in the human hippocampal formation

To analyze the localization and levels of Reelin in the human hippocampal formation we performed an immunoperoxidase staining using Reelin antibodies recognizing the N- (G10 and 142) and C-terminal domains of Reelin (R12/14), to identify putative enrichments in proteolytic fragments of Reelin. Paraffin-embedded sections of ND and AD (n = 8, Table 1) were employed. We detected spherical Reelin-positive structures varying in their size from approximately 60 up to 250 μm^2^ predominantly located in fiber-rich structures (Figure 1 and Additional file 1: Figure S1) in both groups of subjects. They were most abundant in the fornix, enriched in myelinated axons of afferent and efferent projection neurons. A high density of these immunoreactive deposits was also evident in the neuropil of layer I of the pre-/parasubiculum and subiculum. Fewer deposits were detected in layer I of the entorhinal cortex (EC) and the stratum lacunosum moleculare (SLM) of the hippocampus (see Figure 1A for overview). In some cases, Reelin deposits were located in the hilus and rarely also in the pyramidal layer of CA3 and CA1. The immunoreactive signals were specific for Reelin, since control stainings with isotypic IgGs (mouse anti-human CD45) or the omission of primary antibodies did not yield any specific labeling (Figure 1D-E). The control staining using the well-characterized anti-CD45 antibody also indicated that the Reelin-immunoreactivity was not linked to putative invading lymphocytes. Based on their distinct morphology and enrichment in fiber-rich structures, however, we reasoned that they might be related to corpora amylacea (Cam), the age-related spherical bodies suggested to contain a collection of neuronal breakdown products including aggregated proteins and abnormal glycogen bodies [48,49]. Indeed, hematoxylin (according to Ehrlich) and toloidinblue counterstaining identified the Reelin-positive structures as CAm. While all antigen retrieval techniques applied here diminished the Hematoxylin or Toloidinblue stainings, the immunoreactive anti-Reelin signal associated with CAm was never affected (see Methods and Additional file 2: Figure S2).

### Reelin-positive deposits in AD versus non-demented subjects

**Figure 2:**
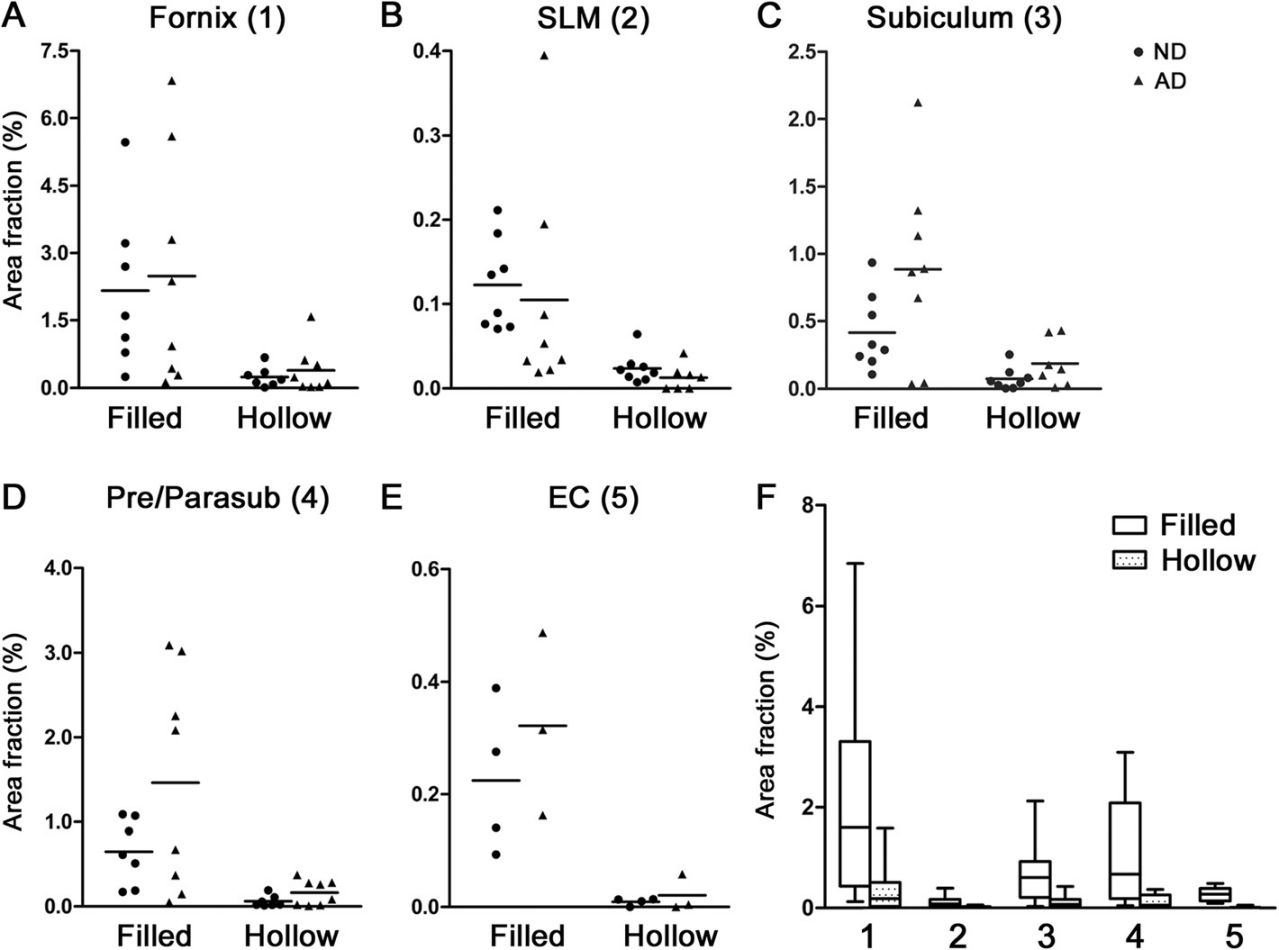
**Summary of stereological analyses of Reelin-positive deposits in ND versus AD individuals. A-E)** Scatter plots of the estimated density of filled and hollow deposits in different areas in the human hippocampus representing the % area covered by Reelin deposits in relation to the total region of interest (AF, Area Fraction). Horizontal lines represent group average. Statistical analysis yielded a significant group effect for the subiculum (F_(1.28)_=4.312, p=0.047), and showed a similar trend for the pre/parasubiculum (F_(1,26)_=3.21, p=0.085). Note that 4 out of 8 ND and 5 out of 8 AD hippocampi blocks were dissected at a level where no EC was present. **F)** Overview of estimated filled and hollow deposit densities across the five brain areas analyzed. Values are displayed in box plot graphs, with the box dimension indicating the 75th (top) and 25th percentile (bottom) and the mean line. The upper and lower error bars indicate the minimum and maximum, respectively. One way ANOVA analysis revealed a significant main effect of deposit type in all areas analyzed that was independent of the dementia status: 1) fornix: F_(1,26)_=11.45, p=0.002; 2) SLM: F_(1,28)_=14.34, p=0.001; 3) subiculum: F_(1,28)_=15.52, p=0.001; 4) pre/parasubiculum: F_(1,26)_=13.52, p=0.001; 5) entorhinal cortex: F_(1,10)_=20.95, p=0.001, statistical significance was set at p<0.05.

With our staining protocol, we were able to identify two different types of Reelin-positive CAm: 1) A filled type; with the entire area of CAm being Reelin immunoreactive (Figure 1B arrow), and 2) a hollow type; with Reelin immunoreactivity being located around the acidic core of CAm (Figure 1B arrowhead). Here, we were interested to determine whether their density differed between ND and AD subjects. We performed a stereological analysis within selected areas of the hippocampal formation (Figure 1). The statistical evaluation of *Area Fraction* (AF) using a one-way ANOVA revealed a significant increase in the area covered by the deposits in AD patients compared to ND controls in the molecular layer of the subiculum (F_(1,28)_=4.31, p=0.047). A similar elevation in deposit area in AD versus NA subjects was seen in the pre/parasubiculum, however, the ANOVA only yielded a statistical trend (F_(1,26)_=3.21, p=0.085; Figure 2C). No statistical group difference regarding the density of Reelin/CAm was evident in the other brain areas (all F<0.93, all p>0.36). The statistical analysis, however, revealed a significantly higher density of the filled compared to the hollow type in all areas investigated (fornix: F_(1,26)_=11.47, p=0.002; SLM: F_(1,28)_=14.34, p=0.001; subiculum: F_(1,28)_=15.52, p=0.001; pre/parasubiculum: F_(1,26)_=13.52, p=0.001; EC: F_(1,10)_=20.95, p=0.001), that was independent of the dementia status (Figure 2). Despite this dominance of filled deposits, the significant increase in total amount of deposits in the subiculum molecular layer in AD was not solely attributed to filled deposits (ANOVA: group × type interaction: F_(1,28)_=2.00, p=0.168) but related to a concomitant increase in both types of deposits.

### Reelin levels in the human cerebral spinal fluid (CSF)

**Figure 3:**
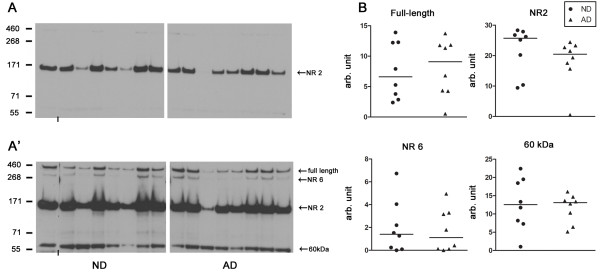
**Reelin levels in the CSF. A)** Anti-Reelin 142 immunoreactive signals on Western blots at low exposure time (40 seconds) to visualize the NR2 fragment. A’) Higher exposure time (18 minutes) of the same blot as A to visualize full-length (~460 kDa), NR6 (~ 310 kDa) and the additional band at ~ 60 kD. Images of immunoblots were cut in half to separate ND from AD for visual display. The short vertical lines at the bottom of the blot indicate joined bands for visual presentation. The HiMark Pre-Stained Standard was used as ladder. **B)** Semi-quantitative analysis of the levels of full-length Reelin and its proteolytic fragments in the CNS. Scatter plots representing relative optical densities expressed in arbitrary units (AU) of CSF samples obtained from ND and AD individuals. Lines represent the group median. Mann–Whitney *U*-test revealed no significant differences between groups.

To assess whether the immunohistochemical findings correlate with the levels of Reelin and its physiologically produced proteolytic fragments in the CSF, previously investigated in AD and ND subjects but yielding conflicting results [36,50,51], we performed a biochemical analysis of the CSF samples collected for each individual of our study. Besides the well-described immunoreactive bands representing full-length Reelin, the NR6 (310 kDa) and NR2 (180 kDa) fragments, we detected an additional band running at around 60 kDa. This recently described novel N-terminal Reelin fragment [47] likely represents the ADAMTS-5-mediated degradation product of Reelin (Figure 3A) that is enriched in aged brain tissue homogenates [47] and detected upon internalization (TN, unpublished findings). Semi-quantitative analysis of two repeated experiments revealed high between-subjects variability and no significant differences regarding the levels of Reelin and the three proteolytic fragments emerged between AD and ND subjects (Figure 3B). However, despite the unknown causal link and the limitations of our sample size, a correlation analyses highlighted a significant relationship between the levels of the 60 kDa fragment and the overall CAm density in subiculum and pre/parasubiculum in ND subjects (subiculum: r=0.857, p=0.004; pre/parasubiculum: r=0.845, p=0.006) that was lost in AD patient (subiculum: r=0.098, p=0.826: pre/parasubiculum: r=0.275, p=0.529), pointing to putative alterations in Reelin proteolytic degradation in AD. None of the other parameters (subfields, Reelin fragments) yielded any significant relationships and no confounding effect of the postmortem interval, pH, brain size or ApoE genotype contributed to this effect.

### Levels of AD-relevant proteins in Reelin-positive CAm

**Figure 4:**
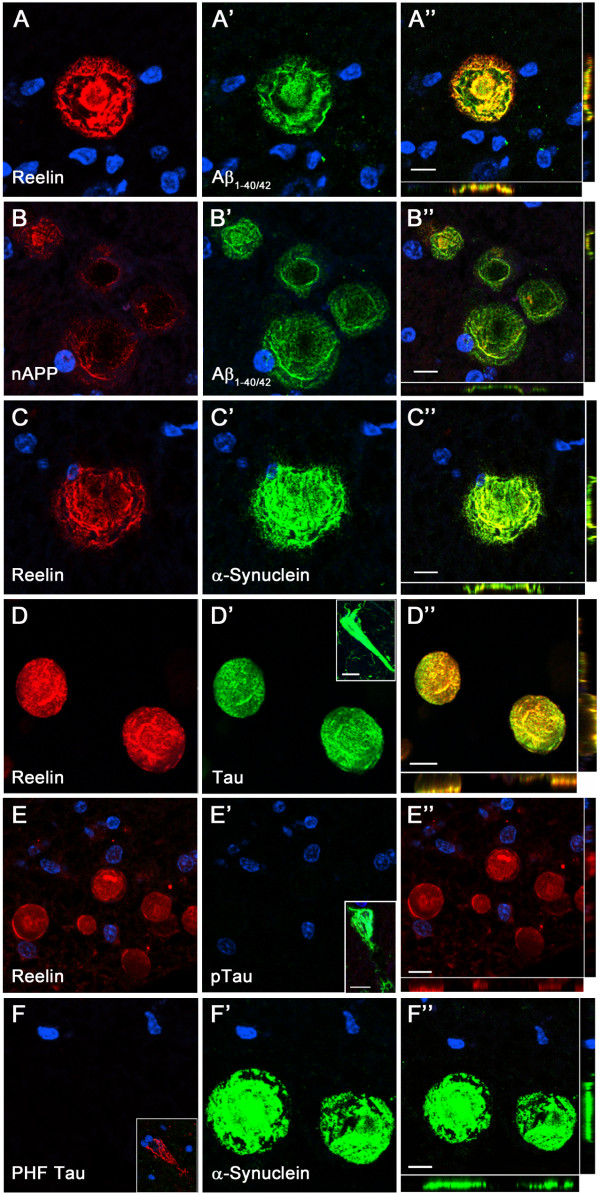
**AD-relevant proteins are present in CAm.** Representative images of double immunofluorescence labeling involving brain sections obtained from an 82 year-old ND individual counterstained with the nuclear dye DAPI (blue). Red channels show the anti-Reelin **(**antibody 142; **A**, **C**, **D**, **E)**, anti-N-APP **(B)**, and anti-PHF Tau (paired helical filament, **F)** stainings. Green channels depict the anti-Aβ_1–40/42_**(A’-B’)**, anti-α-Synuclein **(C’, F’)**, anti Tau **(D’)** and anti-pTau **(E’)** signals. Merged channels are shown in **A**”-**F**”. Inserts in **E**’ and **F** highlight neurofibrillary tangles in CA1 region stained with anti-pTau and anti-PHF Tau antibodies, respectively. Scale bars = 10 μm.

**Figure 5:**
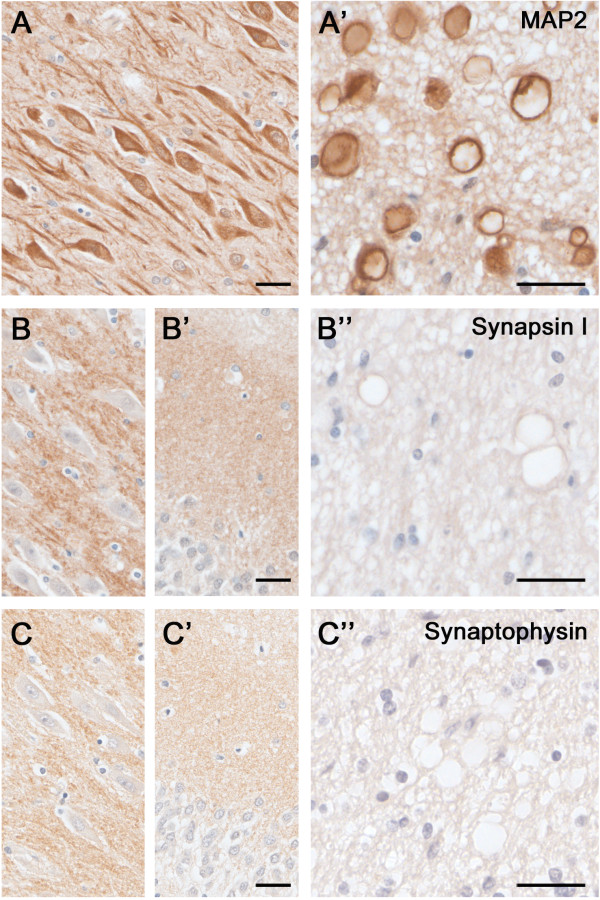
**Dendritic and synaptic proteins in the aged human hippocampus: Differential association with CAm.** Immunoperoxidase-hematoxylin staining of brain sections obtained from an 85 year-old ND individual. **A)** Representative images of the CA2 subfield and fornix **(A’)** labeled with the dendritic cytoskeletal marker anti-MAP2. Note the strong immunoreactivity in pyramidal cell bodies and dendrites, as well as CAm **(A’)**. **B-C)** Immunoperoxidase staining using anti-Synapsin-1 **(B)** and anti-Synaptophysin antibodies **(C)** depicting the CA2 pyramidal cell layer **(B, C)**, the dentate gyrus molecular layer **(B’, C’)** and the fornix **(B”, C”)**. While a characteristic synaptic immunoreactivity of both markers was evident in the hippocampus proper and dentate gyrus, no accumulation of these synaptic proteins was seen in CAm. Scale bars: **A-C** = 30 μm, **A’-C’** = 30 μm.

**Figure 6:**
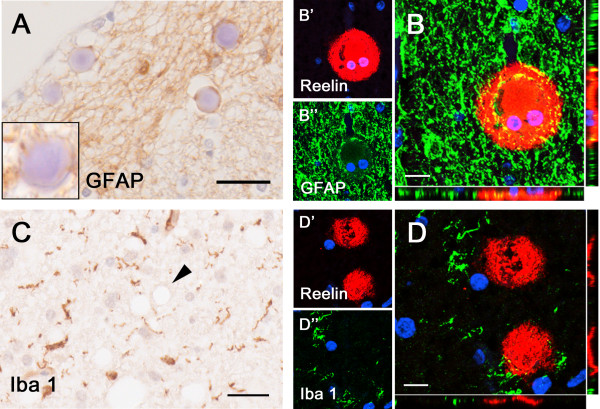
**Association of astrocytes but not microglia with Reelin/CAm deposits.** Immunohistochemical analyses involving brain sections obtained from an 82 year-old ND individual. **A)** Representative image of the fornix labeled with anti-GFAP antibodies and hematoxylin (Ehrlich) staining. Note the GFAP immunoreactivity enclosing the CAm in the dense glial network. Insert represents a Reelin/CAm deposit in an area with low GFAP intensity. **B)** Double immunofluorescence staining with anti-Reelin antibody **(**142, red, **B’)** and anti-GFAP antibody **(**green, **B”)**. Nuclei are visualized with DAPI-enriched mounting medium. **C)** Immunoperoxidase staining using anti-Iba1 antibody and hematoxylin (Ehrlich) counterstaining. The pretreatment of the tissue sections with 10 minute citrate irradiation completely diminishes the CAm labeling (arrowhead). **D)** Double immunofluorescence staining using anti-Reelin antibody **(**142, red, **D’)** and anti-Iba1 antibody **(**green, **D’)**. Scale bars: **A**, **C** = 30 μm; **B**, **D** = 10 μm.

Based on the Reelin-dependent modulation of several AD-relevant pathways, including APP proteolytic processing [52,53] and Tau phosphorylation [8,52], we were interested in determining whether these and other disease-associated proteins were present in the aging-associated Reelin-CAm deposits. Double-immunofluorescence stainings using antibodies raised against the N-terminal domain of APP and β-amyloid sequence 1-40/42 (Aβ_1–40/42_) or anti-Aβ_1–40/42_ in combination with anti-Reelin antibodies revealed their co-localization in most CAm in all areas independent of disease state (Figure 4A). However, the intensity varied depending on the antigen retrieval technique used, in line with previous reports using paraffin-embedded postmortem human brain samples [54,55]. For both, N-terminal APP and Aβ_1–40/42_ antibodies the antigen retrieval methods applied here either strongly decreased the staining intensity (citrate irradiation and pepsin treatment) or completely diminished it (95% FA; Additional file 2: Figure S2) in comparison to non-pretreated sections, suggesting the presence of soluble APP and/or APP fragments rather than Aβ aggregates. Double immunofluorescence stainings using anti-Tau, phosphorylated Tau (pTau) or paired helical filaments (PHF) in combination with anti-Reelin antibodies revealed the presence of Tau, however, no enrichment in phosphorylated Tau species in Reelin-positive CAm (Figure 4, Additional file 2: Figure S2). To confirm and further investigate their origin, we performed double-immunofluorescence staining using additional antibodies recognizing synaptic, axonal and dendritic proteins. In accordance with previous findings α-Synuclein [56], our double-immunofluorescence staining confirmed the presence of α-Synuclein in Reelin-positive CAm. As with the Aβ_1–40/42_ antibodies, the signal intensity dropped upon antigen retrieval technique (Additional file 2: Figure S2). However, anti-Synapsin-1 and anti-Synaptophysin stainings did not show a positive signal of these two abundant synaptic proteins within CAm (Figure 5). On the other hand, the dendritic marker, MAP2, was detected in CAm using immunoperoxidase staining (Figure 5), confirming a neuronal origin and indicating that CAm may originate from both dendritic and axonal compartments. Based on previous findings reporting an association of CAm with astroglia [48], we were interested in determining whether Reelin-positive CAm show a similar relationship to glial cells. To this end we performed an immunhistochemical analysis using antibodies against glial fibrillary acidic proteins (GFAP) to stain astrocytes as well as an anti-Iba1 antibody to label microglia. As reported, the GFAP immunoreactivity was highest in all fiber-rich structures, as well as prominent in glial endfeet enclosing the cerebral vasculature vessels and ventricle walls (Figure 6). In areas with high labeling, GFAP immunoreactivity was found in association with CAm. However, the glial marker was found to be restricted to the border of deposits, but was not associated with any of the other markers that were enriched in CAm, such as APP, Aβ or α-Synuclein. No co-association of Iba1 with CAm was obtained by either immunoperoxidase or -fluorescence stainings. Rather, the staining intensity was homogenous across all areas, indicating that these aging-associated neuronal structures are inert and do not provoke a microglia activation.

## Discussion

Here we describe for the first time the presence of the extracellular matrix protein Reelin within spherical depositions in the human hippocampal formation. Our immunohistochemical stainings revealed the presence of considerable amounts of N- and C-terminus-containing Reelin fragments in these deposits. Stereological analyses indicated that their density in the hippocampal formation is largely independent of the dementia status; however, it revealed a trend towards higher levels in patients with AD and a putative link to alterations in Reelin proteolytic processing. Based on hematoxylin and toluidinblue labeling and their distinct morphology, it is highly conceivable that Reelin and its fragments constitute a major component of CAm, a prominent structure of the aging human brain whose origin and biological significance in the CNS is poorly understood. In the following, we summarize the current status of the CAm literature and discuss the presence and putative role of Reelin in their formation in the human hippocampal formation.

### Aging-associated CAm in the human hippocampal formation

Despite the numerous studies, the role of CAm during aging and neurological diseases has remained obscure. They are commonly seen in the glial feltwork of subpial, subependymal, and perivascular regions, but can also be detected in fiber-rich areas in the CNS, most prominently within the hippocampal formation [48]. The major components of CAm are glucose polymers, presumably linked to aging associated glucose metabolism defects, peptides and proteins (approximately 4%), many of which have been linked to cellular stress responses [56-58]. Several studies indicated that they evolve from neurons, astrocytes or oligodendrocytes due to the presence of cell-specific proteins [48,49,59,60]. However, it is currently unclear whether their glial association is the result of phagocytosis of remnants of degenerated neurons/neurites and vascular metabolites [49,61] or an indicator of glia pathophysiology/degeneration [62]. In line with these data, neurological and neurodegenerative diseases such as temporal lobe epilepsy, multiple sclerosis, and AD are characterized by elevated levels CAm [61,63], and these spherical bodies were shown to correlate with neuronal loss associated with hippocampal sclerosis [63]. The frequent detection of CAm within axons and terminal neurites in the aging ocular system [64], as well as the prominent accumulation of axonal proteins including Tau [56,65], APP [66], and α-Synuclein [56], indicate that they may form in response to cellular stress and/or aging-related impairments in cytoskeletal stability and axonal transport that ultimately lead to axonal swellings, a mechanism recently proposed for amyloid plaque formation in AD [9].

### Reelin accumulates in two types of CAm – link to its expression pattern?

Intriguingly, many of the areas enriched in CAm overlap with the localization of Reelin. This relates to the well characterized cell types in the murine brain that are part of the entorhino-hippocampal, lateral olfactory and retino-collicular tract as well as the parallel fiber system of the cerebellum [67-71]. Furthermore, in vitro studies reported the expression of Reelin in progenitor cells of oligodendrocytes as well as mature oligodendrocytes [72], highlighting the possibility that the Reelin immunoreactivity associated with CAm in fiber-rich areas like the fornix may stem from its production and secretion by progenitors of oligodendrocytes and oligodendrocytes [72]. Studies in primates identified additional areas with Reelin-positive cells showing a wider distribution of Reelin throughout cortical areas than in any other species, indicating that Reelin influences most cerebral circuits in higher mammals [73]. Findings in macaque monkeys showed strong Reelin immunoreactive soma of Cajal-Retzius cells located in layer I, pyramidal neurons in EC layer II, and interneurons of the subicular complex molecular layer (subiculum, pre/parasubiculum), areas which harbor considerable amounts of Reelin-positive CAm. It is conceivable that these cells, shown to degenerate during preclinical stages of AD [74-76] are a major source of Reelin that is detected within CAm. In line with the findings that Reelin is transported along axons and secreted to act on postsynaptic cells [69-71], the deposits located in the SLM, the projection zone of the layer II pyramidal cells, are most likely distal axonal accumulations of Reelin. It is conceivable that aging-associated impairments of axonal stability and hence axonal transport increase the likelihood of accumulations of transported proteins and organelles. This may aggravate during phases of cellular stress that is frequently accompanied by increased levels in damaged and misfolded proteins, which show a much higher propensity to aggregate (for recent review, see [9]). It is therefore plausible, that the decrease in Reelin levels during aging [28,32,33] with its concomitant in- and decrease in Tau [12,52] and n-cofilin phosphorylation [22], respectively, may reduce axonal and dendritic cytoskeletal stabilities and strongly impair transport from and to synaptic sites. In agreement with this hypothesis, our data showed that highly abundant and cellular stress-induced/associated axonal proteins such as APP and α-Synuclein co-accumulate with Reelin in CAm. Our findings that the formic acid pretreatment (used to detect Aβ peptides in amyloid plaques) abolished the anti-Aβ_1–40/42_ immunoreactivity in CAm indicates that soluble APP and/or APP fragments, rather than Aβ peptide aggregates, accumulate in CAm, which is in line with previous reports [66]. The presence of MAP2 in CAm may be indicative of dendritic varicosities or related to missorting of this dendritic cytoskeletal protein into axonal compartments. In support of a putative protein mistargeting, we did not find evidence of phosphorylated Tau species in Reelin-positive CAm, a result that is also in agreement with previous studies [56,65]. This phenomenon may also be linked to potential dephosphorylation of Tau in the putative acidic [8] and/or protein cross-linking and polymerization environment [56], a potential scenario that would also explain the lack of Synapsin-1 and Synaptophysin immunoreactivity in CAm. It is further conceivable that the change in glucose metabolism – potentially linked to aging-related alterations in glial support – promote the formation of abnormal polysaccharide chains that further block axonal transport. The presence of Reelin in the filled type of CAm could be indicative of such an axonal pathophysiology as previously suggested by the findings and discussions in rodents and primates [9,30,77]. However, the detection of Reelin in the hollow type shows that the core of CAm can be formed independently of Reelin and could indicate that this rare type of spheroid might be linked to the accumulation of Reelin in astrocytic endfeet [39]. This is in agreement with the findings shown here and by others that GFAP-positive astrocytes are associated with but not found in the core of CAm [59]. This hypothesis is also in line with our previous findings, demonstrating with 3D-electron microscopy that Reelin-positive varicosities can be extruded from neurites and detected in astrocytic endfeet, potentially related to phagocytosis [30]. The lack of CAm-associated microglia activation reported here is also indicative that these spherical bodies are inert, despite the presence of putative toxic peptides, proteins and cellular metabolites [61]. The distinct association of Reelin at the boarder of CAm – particularly prominent in the hollow type - may be linked to its extracellular secretion and accumulation around CAm that precludes a microglia response. Altogether, this may explain why CAm – despite their close association with aging-associated neurodegenerative processes linked to impairments in glucose metabolism, protein synthesis, transport and degradation – have no major effects on the physiology of neighboring neurons. It would be highly relevant to assess the levels of Reelin associated with CAm localized outside the hippocampal formation (i.e. hypothalamic nuclei), which have been reported to display pronounced cytoskeletal alterations during the course of aging and AD [78].

### Reelin levels and deposits in AD compared to ND individuals

Previous reports indicated that several neurological and neurodegenerative conditions are accompanied by an increase in CAm, potentially linked to higher levels of cellular stress. However, quantitative data are largely missing. Here, we intended to assess the density of Reelin-positive CAm with an unbiased stereological approach involving five distinct areas of the hippocampal formation, complemented with immunoblot analyses to measure the Reelin levels in CSF samples of the same ND and AD individuals. Our stereological analysis revealed that the total amount of Reelin-positive CAm – independent of the type of deposit - was found to be increased in the subicular complex of AD compared to ND individuals. However, due to the small cohort and the high between subject-variability, only the density in the subiculum molecular layer yielded a significant group effect. Despite the limitation due to the small sample size, our data indicate the increase in CAm density may reflect the aggravation the aging-related impairments in cellular functions that result in increased intracellular accumulation of missorted, misfolded or poorly degraded proteins which is known to be increased in AD [9]. It is very likely that putative differences in other areas measured were covered by an aging effect, as there is a clear age-dependent increase in the number of CAm [48]. Currently, it is unknown how these histopathological changes relate to the level of Reelin protein and its proteolytic products in the CSF. While our assessment of full-length Reelin and its proteolytic fragments in the CSF revealed no statistical differences between groups, a correlation analyses highlighted a significant relationship between the levels of the 60kDa fragment and the overall CAm density in the susubiculum and pre/parasubiculum in ND subjects but not AD patients. While a higher sample size is absolutely required to substantiate this finding, the preliminary data indicate that the accumulation of Reelin in CAm might be related to its proteolytic degradation, a phenomenon that appears to be altered in AD versus ND subjects. It will also be highly relevant to assess Reelin levels in CSF and brain tissue homogenates in younger cohorts of ND subjects, as well as MCI and early stages of AD, which is expected to show larger differences to age-matched ND individuals. Differences in the age range of the subjects included in previous studies may have also accounted for the conflicting results regarding the levels of Reelin and its fragments in the CSF published earlier [36,50,51]. It would be therefore highly relevant to investigate the levels of Reelin and all its proteolytic fragments (including the 60 kDa product) and its putative enrichment in neuritic varicosities in a substantially larger cohort of human subjects across aging and AD.

## Conclusion

Altogether, our results indicate that aging- and disease-associated changes in Reelin levels and proteolytic processing might play a role in the formation of CAm by altering cytoskeletal dynamics. Its presence may also be an indicator of a degenerative state of neuritic compartments. However, this remains to be experimentally determined using preclinical models of aging and AD.

## Appendix

### Additional text

We detected the Reelin-positive deposits using various antibodies raised against different Reelin epitopes (Additional file 1: Figure S1A-C). Reelin deposits were stained with all antibodies used, independently of the antigen retrieval technique. To visualize CAm with hematoxylin or toloidinblue, antigen retrieval was omitted in order to maintain the coloration. Figure 1E illustrates the high morphological variations in Reelin/CAm deposits. Besides the prominent staining of Reelin within deposits, we also detected intracellular Reelin immunoreactivity in pyramidal cells of the cornu ammonis, particularly in CA3 and to lesser extent CA1 pyramidal cells, using N-terminal but not C-terminal targeting antibodies (Additional file 1: Figure S1F), in line with a previous report [31]. These immunoreactive signals were, however, only seen using an antigen retrieval approach involving microwave irradiation in citrate buffer followed by pepsin pretreatment (see Methods). Chromatin filters and high magnification microscopy revealed Reelin to be present within cytosolic vesicles (insert, Additional file 1: Figure S1F). To visualize Reelin-expressing cells, which were sparse in our cohort of aged non-demented individuals and shown to further decrease in AD patients [32,79], repeated microwave irradiation in citrate buffer had to be applied (Additional file 1: Figure S1C, insert).

## Competing interests

The authors declare that they have no competing interests.

## Authors’ contributions

All the experimental procedures were carried out by TN. TN and IK wrote the manuscript. Both authors read and approved the final version of the manuscript.

## Supplementary Material

Additional file 1: Figure S1 Immunoperoxidase stainings of paraffin-embedded hippocampal brain sections. **A)** Anti-Reelin immunoreactivity (G10 antibody) in tissue section obtained from a 63 year-old ND individual following antigen retrieval with microwave irradiation in citrate buffer and pepsin pretreatment. **B)** Brain sections of an 89 year-old AD patient, processed for antigen retrieval (citrate and pepsin pretreatment), stained with anti-Reelin (142) antibodies. **C)** Anti-Reelin (142 antibody) immunoperoxidase and hematoxylin staining of tissue section obtained from an 88 year-old ND individual following repeated microwave irradiation in citrate buffer. Insert shows a Reelin-expressing cell located in SLM. **D)** Reelin immunoreactivity (R12/14 antibodies) in tissue section of a ND individual (63 years old) pretreated with citrate and pepsin. **E)** Morphological variations of Reelin-positive deposits located in five brain regions included in the stereological analysis. Representative pictures of immunoperoxidase staining using anti-Reelin antibody (G10) combined with microwave irradiation in citrate buffer and pepsin pretreatments. **F)** Reelin immunoreactivity in pyramidal cells of AD patient (80 years old) visualized with anti-Reelin antibody (G10) following citrate/pepsin pretreatments. **G)** Reelin immunoreactivity (R12/14 antibodies) in tissue section of an AD individual (78 years old) pretreated with citrate and pepsin. Arrowheads point to cytosolic vesicles with immunopositive Reelin labeling. Scale bars: **A**-**D**, **F** =30 μm; **E** = 25 μm. Click here for file

Additional file 2: Figure S2 Antigen retrieval and its effect on staining intensities of AD-relevant proteins in CAm. Representative images of immunofluorescence staining involving brain sections obtained from a ND individual (82 years old) counterstained with the nuclear dye DAPI (blue). Antigen retrieval involved either microwave irradiation in citrate buffer followed by pepsin incubation **(A-B)** or a 95% formic acid (FA) pretreatment **(C)**. **A**) Double labeling using anti-α-Synuclein **(**red, **A)** and anti-Reelin **(**G10, green, **A’)** antibodies, merged in A”. **B)** Anti-Aβ_1–40/42_ antibody **(**red, **B)** combined with anti-Reelin antibodies **(**G10, green, **B’)** show a large degree of overlap **(B”**, merged). **C**) Double immunofluorescence staining using anti-pTau **(**red, **C)** and anti-Aβ_1–40/42_ antibodies **(**green, **C’)**. Note that the FA treatment destroys the anti-Aβ_1–40/42_ signal in the CAm but not in amyloid deposits (arrowhead). The pixel brightness is increased in the merged channels to visualize the presence of the immunonegative deposits **(C”)**. Scale bars = 10 μm. Click here for file
