# Supplementary figures and images for: Reelin immunoreactivity in neuritic varicosities in the human hippocampal formation of non-demented subjects and Alzheimer’s disease patients
Source: Acta Neuropathol Commun. 2013 Jun 26;1:27. doi: 10.1186/2051-5960-1-27 (PMC3893416; doi:10.1186/2051-5960-1-27)

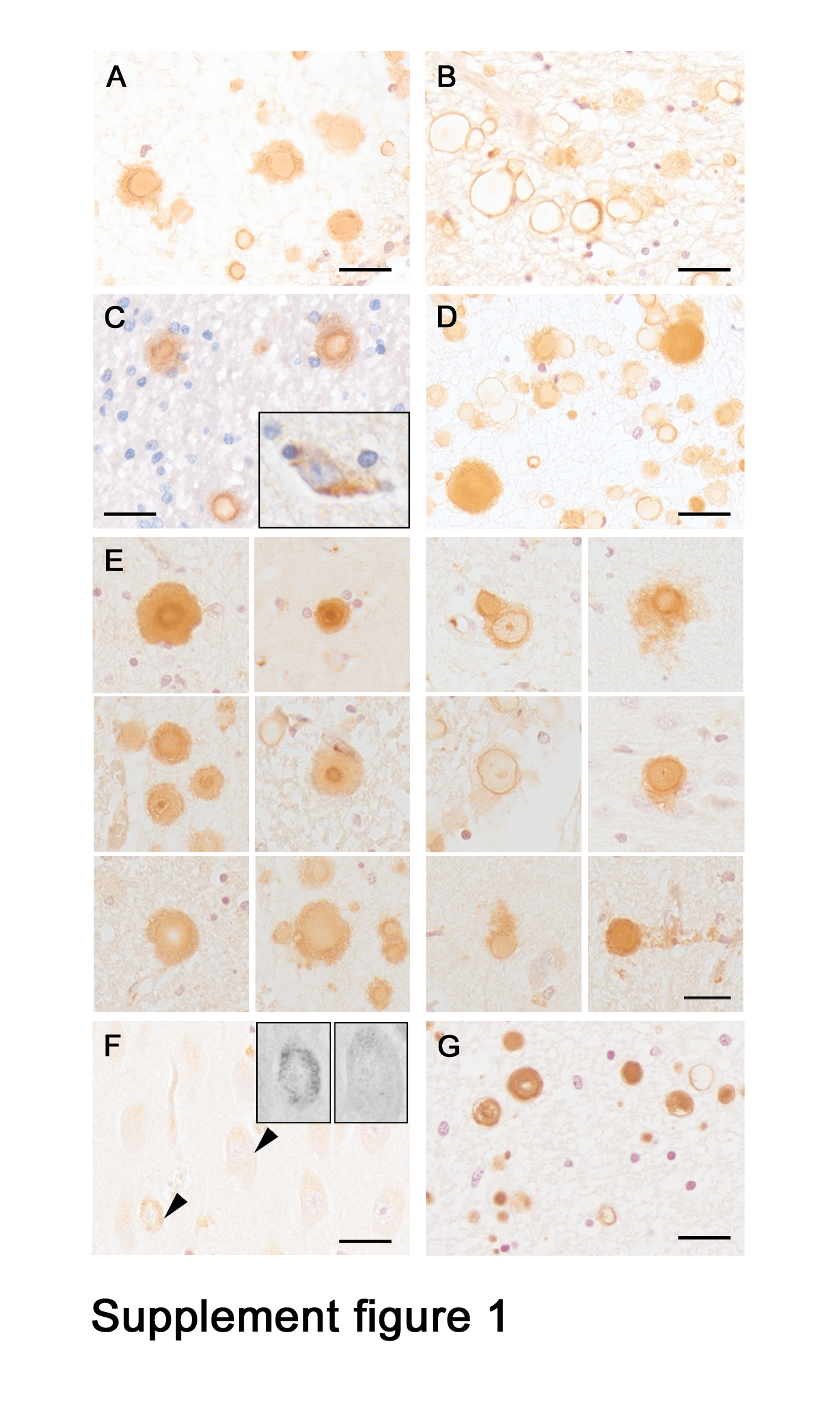

Supplement: Additional file 1: Figure S1 — Immunoperoxidase stainings of paraffin-embedded hippocampal brain sections. A) Anti-Reelin immunoreactivity (G10 antibody) in tissue section obtained from a 63 year-old ND individual following antigen retrieval with microwave irradiation in citrate buffer and pepsin pretreatment. B) Brain sections of an 89 year-old AD patient, processed for antigen retrieval (citrate and pepsin pretreatment), stained with anti-Reelin (142) antibodies. C) Anti-Reelin (142 antibody) immunoperoxidase and hematoxylin staining of tissue section obtained from an 88 year-old ND individual following repeated microwave irradiation in citrate buffer. Insert shows a Reelin-expressing cell located in SLM. D) Reelin immunoreactivity (R12/14 antibodies) in tissue section of a ND individual (63 years old) pretreated with citrate and pepsin. E) Morphological variations of Reelin-positive deposits located in five brain regions included in the stereological analysis. Representative pictures of immunoperoxidase staining using anti-Reelin antibody (G10) combined with microwave irradiation in citrate buffer and pepsin pretreatments. F) Reelin immunoreactivity in pyramidal cells of AD patient (80 years old) visualized with anti-Reelin antibody (G10) following citrate/pepsin pretreatments. G) Reelin immunoreactivity (R12/14 antibodies) in tissue section of an AD individual (78 years old) pretreated with citrate and pepsin. Arrowheads point to cytosolic vesicles with immunopositive Reelin labeling. Scale bars: A-D, F =30 μm; E = 25 μm. [file 2051-5960-1-27-S1.tiff]

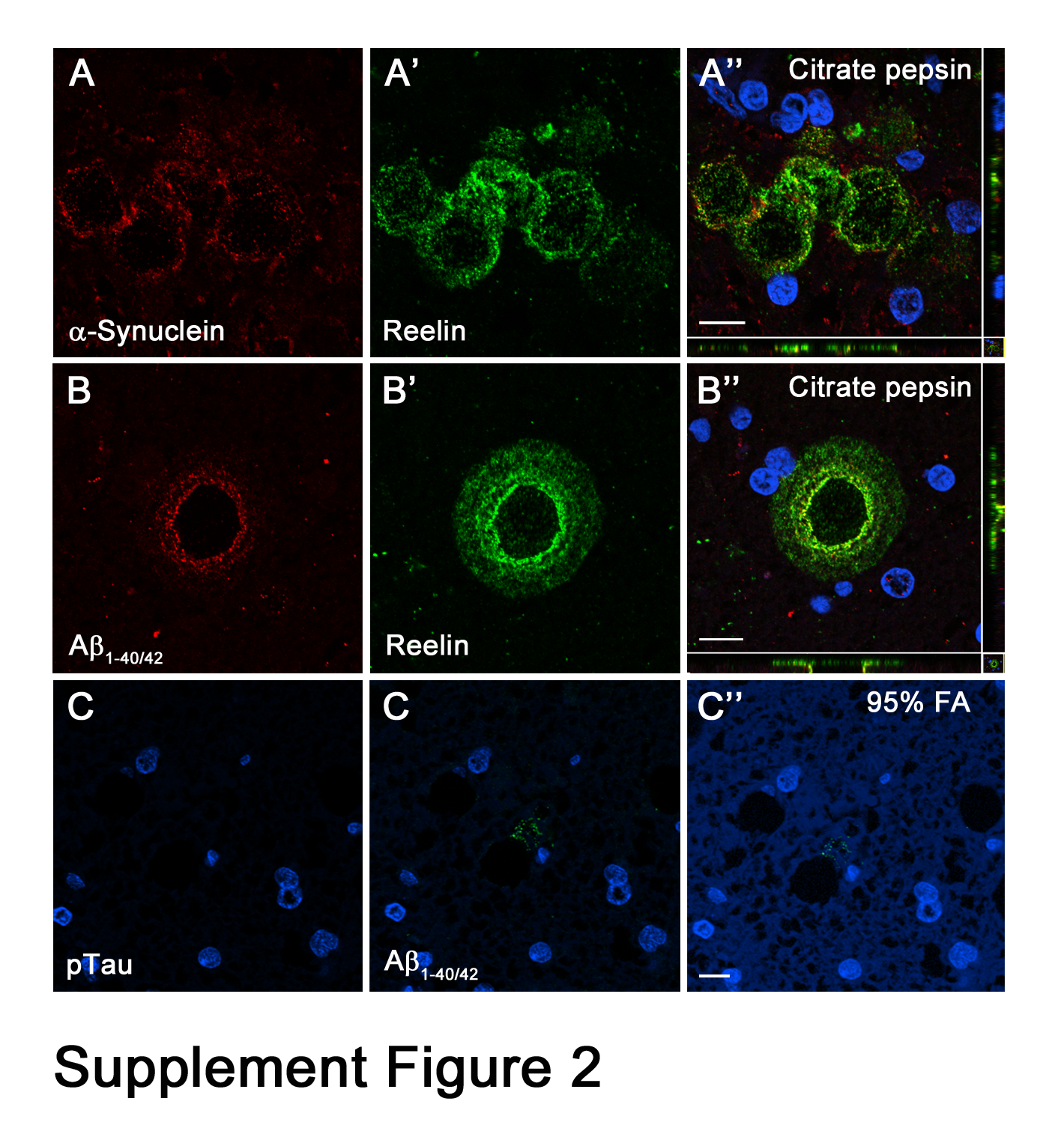

Supplement: Additional file 2: Figure S2 — Antigen retrieval and its effect on staining intensities of AD-relevant proteins in CAm. Representative images of immunofluorescence staining involving brain sections obtained from a ND individual (82 years old) counterstained with the nuclear dye DAPI (blue). Antigen retrieval involved either microwave irradiation in citrate buffer followed by pepsin incubation (A-B) or a 95% formic acid (FA) pretreatment (C). A) Double labeling using anti-α-Synuclein (red, A) and anti-Reelin (G10, green, A’) antibodies, merged in A”. B) Anti-Aβ1–40/42 antibody (red, B) combined with anti-Reelin antibodies (G10, green, B’) show a large degree of overlap (B”, merged). C) Double immunofluorescence staining using anti-pTau (red, C) and anti-Aβ1–40/42 antibodies (green, C’). Note that the FA treatment destroys the anti-Aβ1–40/42 signal in the CAm but not in amyloid deposits (arrowhead). The pixel brightness is increased in the merged channels to visualize the presence of the immunonegative deposits (C”). Scale bars = 10 μm. [file 2051-5960-1-27-S2.tiff]
